# Neurotrophic factors for disease-modifying treatments of Parkinson's disease: gaps between basic science and clinical studies

**DOI:** 10.1007/s43440-020-00120-3

**Published:** 2020-07-22

**Authors:** Piotr Chmielarz, Mart Saarma

**Affiliations:** 1 Department of Brain Biochemistry, Maj Institute of Pharmacology, Polish Academy of Sciences, Krakow, Poland; 2 Institute of Biotechnology, HiLIFE, University of Helsinki, Helsinki, Finland

**Keywords:** Parkinson’s disease, Neurotrophic factors, Translational research, Disease-modifying, Clinical trials

## Abstract

**Abstract:**

**Background:**

Neurotrophic factors are endogenous proteins promoting the survival of different neural cells. Therefore, they elicited great interest as a possible treatment for neurodegenerative disorders, including Parkinson’s Disease (PD). PD is the second most common neurodegenerative disorder, scientifically characterized more than 200 years ago and initially linked with motor abnormalities. Currently, the disease is viewed as a highly heterogeneous, progressive disorder with a long presymptomatic phase, and both motor and non-motor symptoms. Presently only symptomatic treatments for PD are available. Neurohistopathological changes of PD affected brains have been described more than 100 years ago and characterized by the presence of proteinaceous inclusions known as Lewy bodies and degeneration of dopamine neurons. Despite more than a century of investigations, it has remained unclear why dopamine neurons die in PD.

**Methods:**

This review summarizes literature data from preclinical studies and clinical trials of neurotrophic factor based therapies for PD and discuss it from the perspective of the current understanding of PD biology.

**Results:**

Newest data point towards dysfunctions of mitochondria, autophagy-lysosomal pathway, unfolded protein response and prion protein-like spreading of misfolded alpha-synuclein that is the major component of Lewy bodies. Yet, the exact chain of events leading to the demise of dopamine neurons is unclear and perhaps different in subpopulations of patients.

**Conclusions:**

Gaps in our understanding of underlying disease etiology have hindered our attempts to find treatments able to slow down the progression of PD.

**Graphic abstract:**

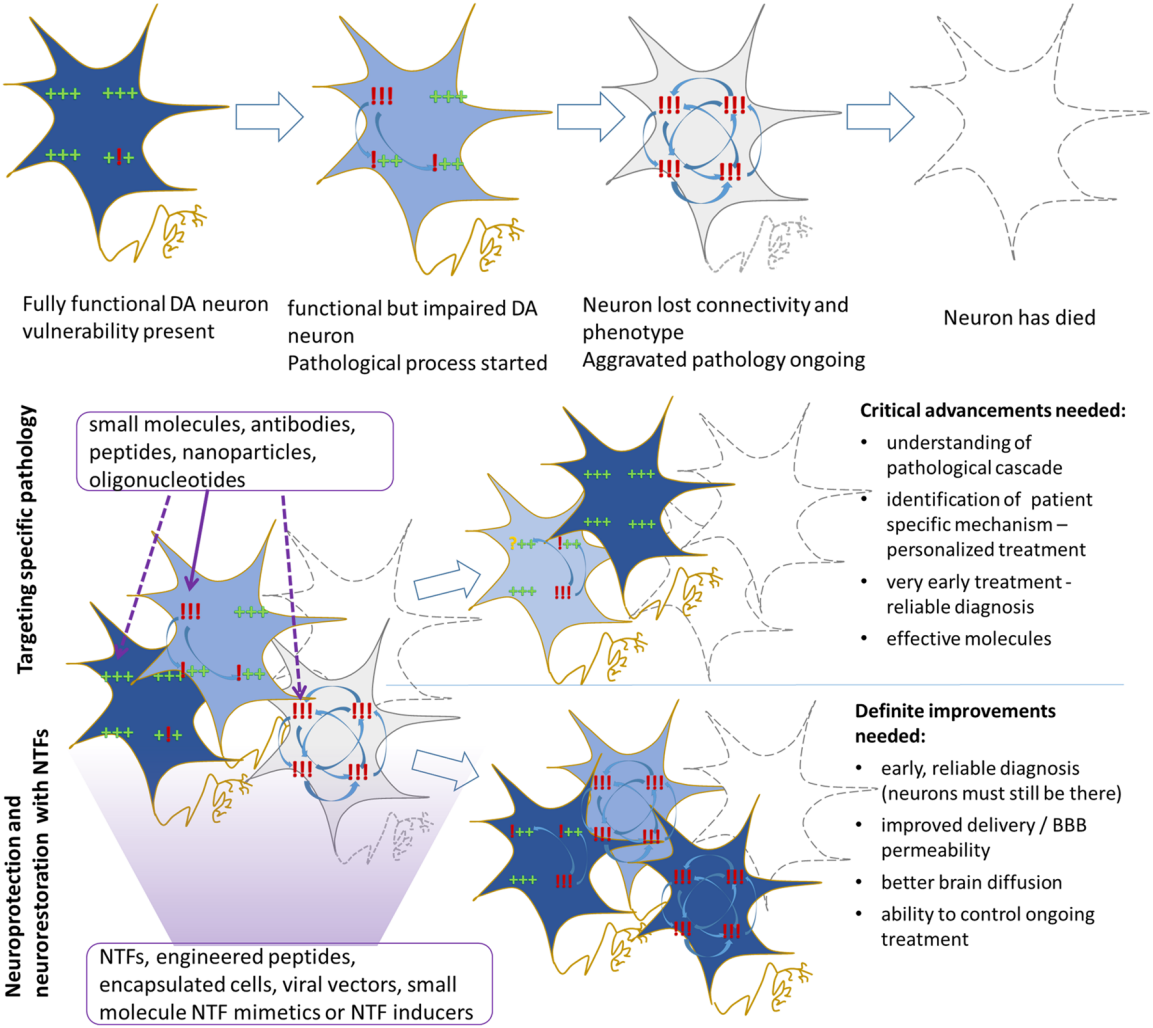

## Introduction

Despite numerous efforts, treatments capable of slowing down the progression of Parkinson’s disease (PD) are still unavailable. Neurotrophic factor (NTF)-based therapies for PD hold great promise, yet they have so far failed to enter the clinic. Technical limitations, such as incomplete delivery protocols, difficulties in selection of the optimal NTF, and its dose, might explain unsatisfactory results with NTFs in PD clinical trials. Furthermore, poor understanding of disease etiology and progression and subsequently, an inadequate clinical trial design, might also have played a significant role. Moreover, our knowledge of the NTFs’ effects on dopamine neurons, especially in the aging, diseased brain, is also limited. In this review, we are aiming to outline the current state of knowledge about PD and molecular processes leading to the degeneration of dopamine neurons and consider implications for the development of disease-modifying therapies for PD. Subsequently, we also summarize preclinical and clinical studies with NTFs in PD, taking into account how they relate to our fundamental understanding of the disease.

### What is Parkinson’s disease?

PD is neurodegenerative disorder affecting about 10 million people worldwide. PD prevalence ranges from 10–1500 per 100,000 depending on the population. The highest prevalence is in Europe and North America and the lowest in Africa and Asia [1] with a higher incidence in males than females [1]. The most significant risk factor for PD is age [1]. Other reported risk factors include pesticide exposure and rural living [1, 2]. Additionally, about 5–10% of PD cases are familial forms linked with chromosomal regions named in chronological order *PARK1, PARK2, PARK3*, etc.[2]. Monogenic forms of PD account for 3–5% of total PD cases. They are caused by mutations in more than a dozen genes such as *SNCA* (*PARK1/4*) (encoding α-synuclein), *Parkin* (*PARK2*), *PINK1* (*PARK6*), *DJ-1* (*PARK7*), *LRRK2* (*PARK8*), *ATP13A2* (*PARK9*), *VPS23C* (*PARK23*), etc.[2]. Moreover, mutations in *GBA* gene encoding β-glucocerebrosidase (GBA) is the greatest genetic risk factor for PD [2, 3].

Classic symptoms of PD are motor abnormalities like resting tremor, bradykinesia, postural instability, and muscle rigidity [2], caused by the death of dopamine neurons in the substantia nigra pars compacta (SNpc). This neuronal loss begets dopamine deficiency in the basal ganglia leading to observed abnormalities in motor functions. Dopamine deficiency regulation is also the main target of currently available treatments either through promoting the synthesis of dopamine, inhibiting dopamine degradation, or direct activation of dopamine receptors. Such symptomatic treatments of PD are very effective in the early and middle stages of disease progression [2]. However, the underlying neurodegeneration advances slowly but inexorably, leading to worsening of the patients' condition, decreased responsiveness to symptomatic treatment, eventually causing death preceded by phase with severe frequent falls, severe dementia and hallucinations [2]. Knowing that death of SNpc dopamine neurons is a prominent neuropathological feature of PD, it was not surprising that neurotrophic factors (NTFs), with their strong survival-promoting action on neurons, elicited high hopes for the neuroprotective treatment. However, while initial preclinical studies demonstrated excellent efficacy of trophic factors in animal models, none of these treatments have so far entered the clinic [4].

### Motor and non-motor symptoms of PD

Motor symptoms have been associated with PD since its initial description more than 200 years ago and still constitute the bulk of PD diagnosis criteria [5]. These are bradykinesia (slowness in the initiation of motion), rigidity, resting tremors, and postural instability. Apart from motor features, it is increasingly recognized that a plethora of non-motor symptoms are occurring in PD. These include constipation, sleep disturbances, anosmia, and increased incidence of depression [2]. In later stages of the disease progression, additional symptoms such as cognitive impairment, fatigue, pain, orthostatic hypotension, and urinary problems develop [2]. Importantly, some of the aforementioned non-motor symptoms precede motor symptoms, and their diagnosis indicates the pre-symptomatic phase and progressive character of the disease. The linkage between non-motor features of PD and degeneration of dopamine neurons remains, in most cases, unclear. Non-motor symptoms do not respond to dopamine replacement therapies [2], and conversely, it is unknown if they will be alleviated by NTFs or other disease-modifying therapies aimed to increase SNpc dopamine neuron survival. However, gastrointestinal problems have been linked to the loss of enteric dopamine neurons and the presence of Lewy pathology in the enteric nervous system [6]. Interestingly, cerebral dopamine neurotrophic factor (CDNF)-deficient mice develop an age-dependent loss of enteric neurons that occurs selectively in the submucosal but not in the myenteric plexus. The deficits in enteric neurons are reflected functionally in delayed gastric emptying, slowed colonic motility, and prolonged total gastrointestinal transit. The selective vulnerability of enteric neurons to the absence of CDNF is reminiscent of the tendency of pathological abnormalities to occur in the gastrointestinal tract in biopsies of patients with PD [7]. Moreover, GDNF and NRTN also have a prominent effect on the development and maintenance of enteric neurons [8].

### Progression of Parkinson’s disease

**Fig. 1:**
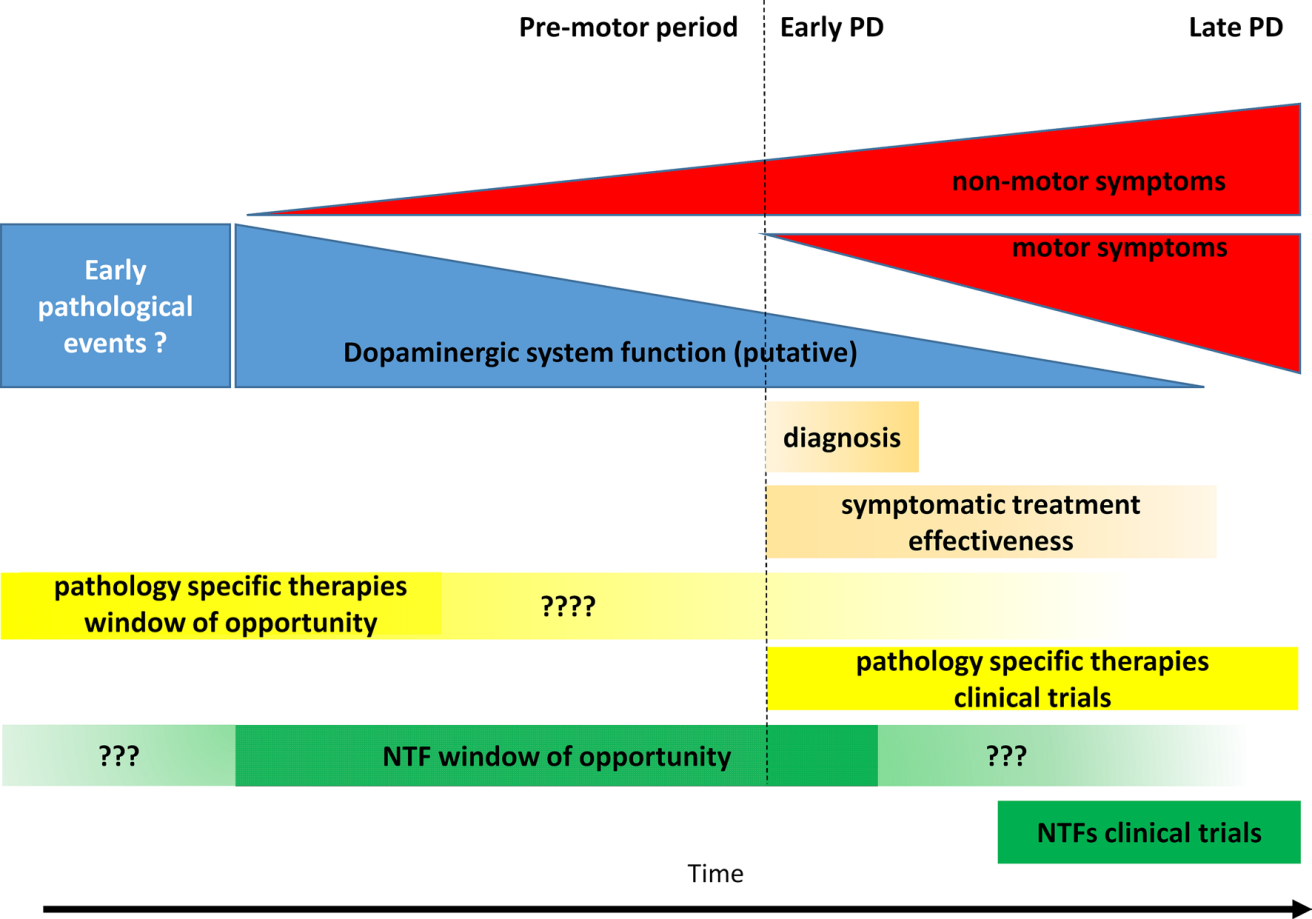
Approximate time course of Parkinson’s disease (PD), putative treatment effectivness and clinical trial schedules. Age is the strongest risk factor for PD and accumulating pathological events in dopamine neurons probably starts decades before the onset of the disease. Similarly, non-motor symptoms are present as long as 20 years before the diagnosis of PD. At the time of motor symptoms and diagnosis, an already significant portion of dopamine neurons is lost. Symptomatic treatment enhancing the action of remaining neurons is still effective for several years, however these remaining neurons rapidly degenerate, and the effectiveness of the treatment diminishes. Based on preclinical data, NTF-based therapy would also be most effective immediately after diagnosis based on motor symptoms, or even better if administered in the presymptomatic phase, however actual clinical trials were performed in mid-to-late stage patients due to ethical issues

It is currently believed that a complex interplay between environmental factors and genetic predispositions is responsible for PD development. The age seems to be the strongest risk factor of the disease [1]. However, it is now commonly accepted that PD starts many years before its clinical diagnosis [2]. Motor symptoms, which are the hallmark of PD, manifest when degeneration and loss of dopamine neurons are already reaching at least 30%, albeit some estimates are as high as 60% [9, 10]. Neurodegeneration progresses rapidly after the initial diagnosis, with majority of the SNpc dopamine neurons lost within 4–5 years [9]. Advanced neurodegeneration at the time of diagnosis and its fast pace afterward has far-reaching implications for the development of treatment strategies for PD. Drugs that would slow down or stop the neurodegeneration should be administered immediately after diagnosis, or preferentially even during the presymptomatic period. The prodromal period, before the onset of motor symptoms and implicitly before significant loss of dopamine neurons, can be as long as ten years or even more [2, 10]. This time could be a window of opportunity where treatments, even modestly slowing down the pace of neurodegeneration, could have a significant impact on treatment outcome. However, actual clinical trial design for NTF-based therapies has followed the opposite strategy. NTFs were administrated mainly to relatively late-stage patients (Fig. 1), to minimize the risk involved with complicated brain surgery and because of the problems related to the diagnosis of PD. Early diagnosis of PD is not trivial. Its base is mainly on the presence of bradykinesia and at least one of the other motor features (rigidity, resting tremor, and postural instability) [2]. Importantly, these symptoms are not specific, and other underlying conditions have to be excluded, while additional criteria such as responsiveness to dopamine replacement therapy and progressive character support a diagnosis of PD. Confidence in diagnosis is often gained only years after initial motor symptoms are observed. Traditionally, confirmation of PD diagnosis can be obtained by histopathological examination of post-mortem brain samples by finding Lewy bodies and assessing dopamine neuron degeneration [2, 11]. Quantification of dopamine neuron degeneration is also possible through single photon emission computed tomography (SPECT) or positron emission tomography (PET), however these expensive imaging techniques are not yet routinely used in the clinic for PD diagnosis [2].

### Histopathological features of PD

The healthy human brain has in both hemispheres in the SNpc about 0.5 million dopamine neurons. The main pathological finding in the PD affected brain is the loss of dopamine neurons in the SNpc and their projections to caudate putamen [11]. Patients with an initial diagnosis already show significant degeneration and loss of these neurons [9, 10]. While dopamine neurons in the SNpc have been the main target of treatments aimed to stop neurodegeneration, it is important to remember that other brain areas are also affected. Neurodegeneration linked with PD is observed in the amygdala, hypothalamus, raphe nucleus, and locus coeruleus [12]. It is currently unclear how neurodegeneration regions other than the SNpc area contribute to the motor and non-motor symptoms or whether it could affect the degeneration of dopamine neurons. Although it was suggested that the loss of noradrenergic stimulation from locus coeruleus could make dopaminergic cells more prone to degeneration [13].

The second characteristic histopathological feature of PD is the presence of Lewy pathology (LP) in the form of insoluble proteinaceous aggregates found in neuronal soma (Lewy bodies) and processes (Lewy neurites) [14]. The protein α-synuclein was demonstrated to be the main component of these aggregates [14]. Spreading of LP through the brain was proposed to correspond to clinical stages of the disease [15], and curiously, it seems to follow neuronal connections [12]. There is mounting evidence that α-synuclein can spread through neuronal networks in a prion-like fashion [16].

However, while Lewy pathology has convincingly been shown as a biological marker of PD, its causative role is far from being proven. Actually, in a subset of patients with familial forms of PD, LP was not found in post-mortem brain examinations [17]. Moreover, Lewy bodies and Lewy neurites can also be found in some patients without diagnosed PD [5]. The process of LP formation, and their contribution to the degeneration of dopamine neurons, is yet to be fully elucidated. Nonetheless, ways of stopping transmission of α-synuclein, and thus preventing the spreading of LP, are intensively pursued in the hope that they would stop disease progression.

Another pathological feature found in PD patient brains are signs of ongoing neuroinflammation i.e., markers of reactive astrocytes and microglia [18], indicating a non-cell autonomous mechanism involved in neuronal death in PD. Through the release of pro-inflammatory cytokines and reactive oxygen species, reactive astrocytes and microglia could be directly damaging dopamine neurons. Alternatively, triggering their inflammatory activation could lead to decreased neurotrophic support they normally provide [18]. However, it remains unclear whether neuroinflammation is a proximal or distal phenomenon, how significant is its contribution to dopamine neuron demise, and if it might also have some protective role.

### Heterogeneity of PD

Adding to the complexity of PD is the fact that both motor and non-motor symptoms of PD exhibit heterogeneity among patients. There have been several attempts to classify different subtypes of PD, but until now, no general agreement has been made [19]. Subtypes of PD have different rates of disease progression and might not share the same etiology [20]. Such a notion is in line with a multitude of diverse environmental and genetic risk factors for PD. Understandably, this might have major implications for clinical trial outcomes, suggesting that PD patients might require a more personalized approach, targeting treatment to specific pathological processes which could drive PD progression only in a subset of patients [21].

### Clinical features of PD and disease-modifying treatments

Altogether, the existence of premotor symptoms up to 10 years before diagnosis and the loss of a significant amount of dopamine neurons reported at the onset of motor symptoms imply advanced neuropathology already exists in what we call early PD. Moreover, loss of majority of dopamine neurons occurs within 5–7 years after diagnosis. Importantly, we do not know how advanced is the pathology at the cellular level in the remaining neurons at the time of diagnosis. These neurons may already be past a stage where targeting a single pathological process could restore cellular homeostasis. Similarly, drug trials targeting a single pathological mechanism would also be doomed to fail in a general patient population if PD heterogeneity is reflecting different underlying etiologies. In fact, multiple clinical trials targeting oxidative stress, which is widely accepted to be involved in PD, failed despite being tested in early-stage patients. The currently ongoing MOPES-PD trial (NCT02906020) targets the patient-specific mechanism (i.e. impaired glucocerebrosidase activity) in early stages of familial forms of PD. If successful, it would be strong support for patient-specific early-stage treatments. However, for personalized treatment of most patients, a test capable of distinguishing patient subpopulations, preferably at the pre-motor stage, would be required. Interestingly, monoamine oxidase (MAO) inhibitors rasagiline and selegiline have demonstrated modest disease-modifying effects when administered to early-stage patients [22, 23]. MAO inhibitors seem to exert protective effects on dopamine neurons through multiple mechanisms, including stimulation of NTF signaling providing general survival-promoting cues [22]. However, this effect could not be fully disentangled from symptomatic effects, and the compounds are not officially recognized as disease-modifying agents [23].

At the same time, it is becoming increasingly clear that in late-stage PD patients, there is little hope for effective treatment, regardless if it is targeting specific pathological mechanisms or having a non-specific survival-promoting effect on neurons. Mostly due to the technical complications and resulting ethical concerns, NTF clinical trials for PD had been tested in mid-to-late stage patients unsuccessfully.

Apart from the quickly progressing demise of dopamine neurons it is worth considering that progressing pathology is not limited to SN dopamine neurons as exemplified by degeneration of noradrenergic neurons in locus coeruleus and the widespread presence of pathological protein aggregates throughout PD patient brains. We do not know how much lasting improvement we can achieve if we try to rescue only dopamine neurons. In fact, it has even been suggested by pre-clinical data from us and others that loss of noradrenergic neurons might increase the susceptibility of dopamine neurons in the SN [13]. Furthermore, most non-motor symptoms of PD are unresponsive to dopamine replacement therapies suggesting that they arise from non-dopaminergic pathology. Therefore, non-motor symptoms will probably continue to progress even if we manage to stop degeneration of dopamine neurons successfully.

## Disease etiology

Although PD symptoms and some treatments have been described in ancient Chinese and Indian documents even 300–400 years BC, scientifically, PD had been described more than 200 years ago, and for more than 100 years we have known that progressive degeneration of dopamine neurons is underlying its motor symptoms [24]. Yet, we still do not understand the molecular cause of their demise, and we know even less about the degeneration of other vulnerable populations of neurons.

### Unique properties of dopamine neurons

Premature death of dopamine neurons in PD might be linked to their unique properties. First, the use of dopamine as the main neurotransmitter has been proposed as a potential liability. Dopamine easily oxidizes, and any defects in dopamine storage inside intracellular vesicles could lead to oxidative stress and, subsequently, mitochondrial and lysosomal dysfunction [25]. Another feature of dopamine neurons are very long and highly branched axons [26]. In mice, a single dopamine neuron innervates ~ 75,000 target neurons with a total length of axons in the striatum of almost 50 cm [26]. Therefore, activity of dopamine neurons requires a large amount of energy and maintaining a high level of intracellular trafficking and protein translation, especially of synaptic proteins such as α-synuclein. Moreover, the most vulnerable dopamine neurons in SNpc exhibit constant autonomous pacemaking activity [12]. It has been proposed that the reliability of dopamine neuron firing is critical for the survival of an organism because it is involved in the rapid motor response to environmental challenges [12]. Therefore, evolution would favor reliability of firing even at the cost of faster “wear off” of cellular components. Accordingly, it would be more important to maintain high ATP production, even at the cost of occasionally generating increased oxidative stress. Similarly, maintaining protein translation would take precedence over maintaining protein homeostasis. In the long run, this could lead to an accumulation of damage and stochastic degeneration of individual neurons. Indeed dopamine neurons seem to degenerate throughout our lifespan [10]. Because the threshold for the manifestation of motor PD symptoms is the loss of about 60% of dopamine neurons, an age-dependent loss would not be a problem during the lifespan of most people. However, a lower initial number of neurons, increased rate of accumulating damage, or an additional pathological trigger putting strain on an already vulnerable and diminished population of dopamine neurons could precipitate the onset of PD [10].

### Mitochondrial dysfunctions

Combined with the enormous synaptic network, autonomous pacemaking puts considerable energetic demands which strain mitochondria, potentially leading to mitochondrial damage and oxidative stress. Moreover, SNpc dopamine neurons lack Ca^2+^ buffering capacity [27], and their pacemaking activity is linked with large Ca^2+^ fluctuations [28]. These Ca^2+^ fluctuations modulate membrane potential and stimulate mitochondrial oxidative phosphorylation to maintain the required energy levels [29]. One side effect of pacemaking activity and Ca^2+^ fluctuations in dopamine neurons is metabolic stress, which in the long run could lead to mitochondrial and lysosomal dysfunctions [29]. Support for the involvement of high calcium levels and mitochondrial dysfunction in PD is the fact that several PD-related genes are implicated in mitochondrial maintenance and turnover, and antioxidant defense (DJ-1, PINK1, Parkin) [2]. Additionally, damaged mitochondria and Ca^2+^ fluctuations can affect the capacity of cells to deal with misfolded proteins [12, 30], which could make them more vulnerable to Lewy pathology. Conversely, Lewy pathology, or specifically the accumulation of misfolded or aggregated α-synuclein, was shown to impair mitochondrial function, autophagy, and calcium balance [31], potentially forming a vicious cycle.

### Autophagy-lysosomal pathway dysfunction

Dysfunctions in cellular degradative pathways that recycle unwanted or faulty cellular components like proteins (i.e. chaperone-mediated autophagy and ubiquitin–proteasome system) and organelles (i.e. macroautophagy) have also been implicated in PD pathology [32]. Mutations in PD-related genes *LRRK2* and *DJ-1* were shown to impair the autophagy-lysosomal pathway [33] and a single allele mutation in the *GBA* gene encoding lysosomal enzyme GBA increases PD risk by five times [3]. Also, aggregated α-synuclein has been shown to impair the autophagy-lysosomal pathway [31]. Reciprocally, abnormalities in protein degradation pathways can lead to increased accumulation of misfolded α-synuclein [34]. A similar feedback loop might happen between mitochondrial dysfunction and macroautophagy since oxidative stress was reported to impair the latter, while impaired macroautophagy would be detrimental for maintaining a healthy mitochondrial pool by slowing mitochondrial turnover [35].

### ER stress and the unfolded protein response

**Fig. 2:**
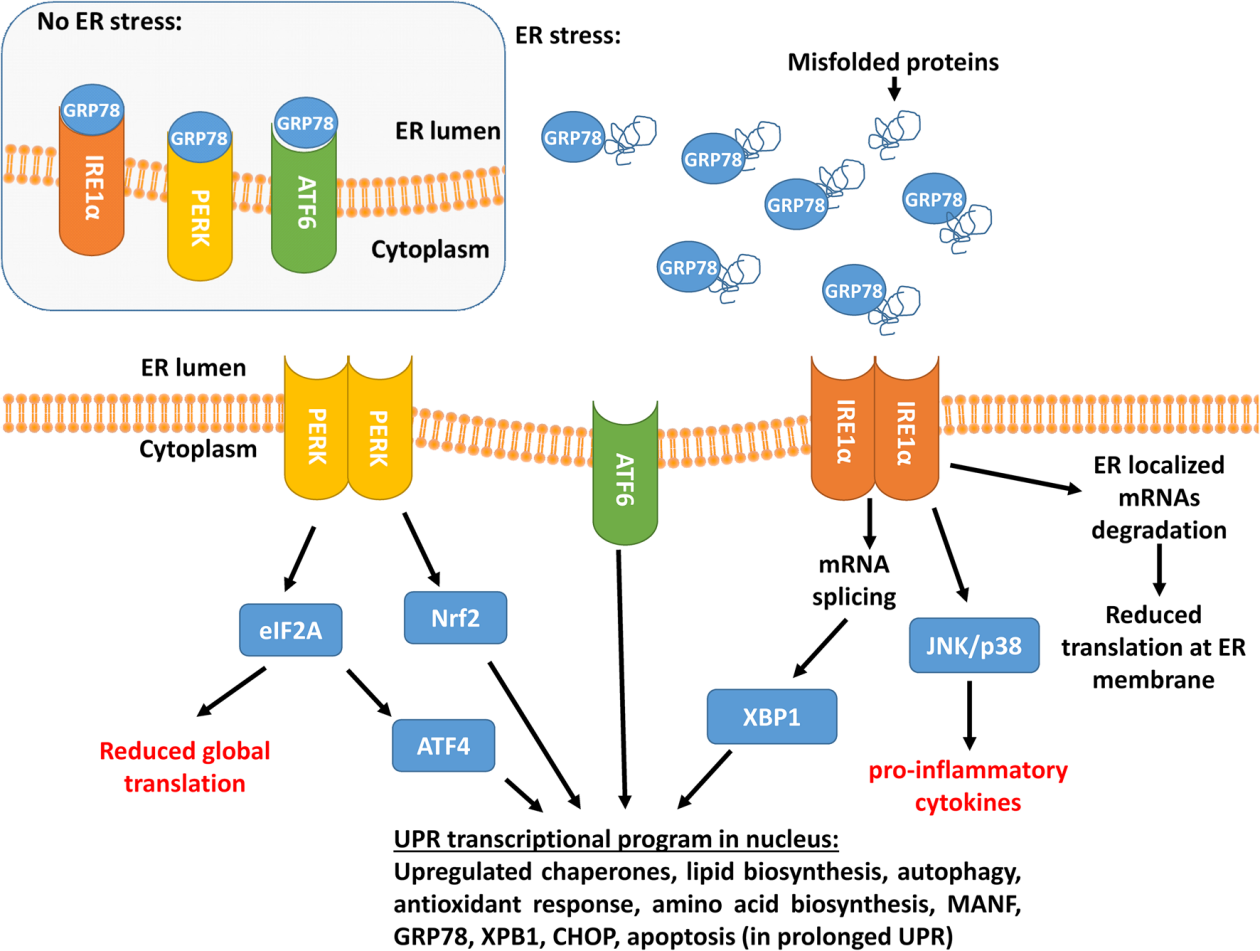
ER stress and unfolded protein response (UPR) pathways. Activation of the unfolded protein response (UPR) is, in a normal state, prevented by interaction of GPR78 with UPR sensors inositol-requiring protein 1 (IRE1α), protein kinase RNA-like ER kinase (PERK) and activating transcription factor 6 (ATF6). Accumulation of misfolded proteins leads to dissociation of GPR78 from the ER membrane located UPR sensors and activation of the UPR. Activated ATF6 translocates to the nucleus where it acts as a transcription factor. IRE1α, a transmembrane serine-threonine kinase and endoribonuclease, degrades ER-localized mRNAs and splices mRNA of X-box binding protein 1 (XBP1) transcription factor, increasing its expression. In chronic and severe ER stress, hyperoligomerized IRE1α recruits TRAF2 and ASK1 by its cytosolic domain and this complex triggers apoptosis via p38 MAPK and JNK pathways followed by enhanced transcription of pro-inflammatory genes. PERK phosphorylates eukaryotic translation initiation factor 2α subunit (eIF2α), reducing translation of most genes, concomitantly leading to increased translation of activating transcription factor 4 (ATF4). Moreover, PERK directly activates erythroid 2-related factor 2 (Nrf2) transcription factor. Together activated transcription factors increase expression of genes involved in protein folding, lipid biosynthesis, protein degradation, antioxidant response, and the UPR itself while the translation of other genes is reduced. Persistent block on translation can be detrimental to neurons. Additionally, if prolonged, UPR leads to upregulation and activation of pro-apoptotic genes

Another important process linked with maintaining protein homeostasis and implicated in PD is endoplasmic reticulum (ER) stress and activation of unfolded protein response (UPR) pathways [36]. ER stress and the UPR are cellular responses which strive to restore proteostatic balance by limiting new protein synthesis and increasing both chaperone activity and misfolded or aggregated protein degradation [36]. While initially protective, UPR, if protracted, is detrimental especially to neuronal cells and can lead to apoptotic cell death. The UPR is activated upon an accumulation of misfolded proteins in the ER lumen and their interaction with ER chaperone GPR78 protein (also known as binding immunoglobulin protein (BiP)). This leads to the dissociation of GPR78 from the ER membrane, where it normally binds three sensors of the UPR, triggering their activation (Fig. 2). These three UPR sensors are inositol-requiring protein 1 (IRE1α), protein kinase RNA-like ER kinase (PERK), and activating transcription factor 6 (ATF6). IRE1α is the ER transmembrane serine-threonine kinase and the endoribonuclease degrading or splicing mRNAs near ER. Activated ATF6 acts as a transcription factor itself, while PERK phosphorylates eukaryotic translation initiation factor 2α subunit (eIF2α), reducing global translation except for specific mRNAs [36]. The transcription factor nuclear factor erythroid 2-related factor 2 (Nrf2) is the second PERK substrate, and it is a critical effector of PERK-mediated cell survival. Together these three arms of the UPR lead to downregulation of global protein synthesis with simultaneous upregulation of genes involved in protein folding, lipid biosynthesis, and protein degradation. Persistent activation of UPR leads to upregulation and activation of pro-apoptotic genes like transcription factor C/EBP homologous protein (CHOP), activation of apoptosis signal-regulating kinase 1 (ASK1) and Nuclear Factor Kappa-light-chain-enhancer of activated B cells (NF-κB) and inhibition of anti-apoptotic B-cell lymphoma 2 (BCL-2) family members [37]. Moreover, prolonged reduction of protein synthesis can be especially detrimental to neuronal function [38].

Activation of the UPR has been demonstrated in post-mortem PD patients' brains [39] and linked with the accumulation of misfolded α-synuclein [40], which can localize to the ER [41]. ER stress is also induced by many neurotoxins interfering with mitochondria action and used to model PD [42], suggesting a link to mitochondria dysfunction.

### α-synuclein and Lewy pathology

Multiplications of the α-synuclein gene cause a familial form of PD [43], supporting α-synuclein’s pathological potential in humans. We know that α-synuclein can exist in several different conformers in monomeric or oligomeric forms, as well as in fibrils of different sizes and conformations [44]. Preclinical studies investigating the role of α-synuclein in PD utilized overexpression of α-synuclein [45] or more recently seeding of α-synuclein misfolding and aggregation with prion-like preformed α-synuclein fibrils (PFFs) [46] both in vitro and in animal models [16, 46]. In these models, α-synuclein overexpression, misfolding and/or aggregation was linked with dysfunctions of mitochondria, autophagy, intracellular transport, neurotransmitter release, disturbances of protein homeostasis, ER stress, and UPR [31, 47], and more recently with compromised DNA repair [48]. Recently, expression of aggregation-prone truncated 1–120 α-synuclein fragment caused evident, progressive neuronal loss accompanied by motor abnormalities [49]. However, significant cell death has not been consistently observed in many of the preclinical models based on α-synuclein overexpression [45]. Also, mice injected with PFFs, which induced profound aggregation of endogenous α-synuclein, have only demonstrated limited cell loss 180 days after PFF injection [16]. Only by simultaneous overexpression of α-synuclein in SN and inoculation with PFFs, a more robust phenotype demonstrating significant dopamine neuron loss was observed [50]. Moreover, it remains an open question how well artificially induced α-synuclein misfolding and aggregation can model LP observed in PD patients. Furthermore, the causative role of LP in PD patients has not been proven. Clinical data reveal a correlation between LP density and dopamine neuron loss [51]. However, when analyzing brains with early LP outside the SNpc, and without PD symptoms (putatively pre-symptomatic), a cell loss of up to 20% of SNpc dopamine neurons was found, despite the lack of LP in SNpc [51, 52]. Explanation of cells loss in SNpc devoid of LP might be that neuronal death was caused by misfolded α-synuclein monomers or oligomers [53], while LP is an epiphenomenon or cell attempt to sequester aggregated proteins. In contrast, two recent studies suggest that the LP formation and sequestration of intracellular organelles is driving neuronal death, rather than α-synuclein itself. Shahmoradian et al. [54] used Correlated Light-Electron Microscopy to evaluate the composition of LP in post-mortem human brains. Apart from α-synuclein, LPs were found to contain a large number of membranous structures, fragments of lysosomes, and mitochondria. An important role of membranous components of LP was corroborated by a recent PFFs-based in vitro study [55]. The authors demonstrated that, triggered by PFFs, aggregates of endogenous α-synuclein transformed into Lewy body-like structures 2–3 weeks after seeding. At this time, they started to include membranous components akin to human LP. Proteomic analysis of these mature aggregates demonstrated enrichment of proteins belonging to mitochondria, ER, Golgi, endolysosomal pathways, and synapses [55]. Interestingly, partially damaged mitochondria present in these aggregates were capable of increasing oxidative stress and activating cell death pathways. There have also been enrichment in proteins linked with the ER stress response, suggesting that the UPR pathway might also be dysregulated. Importantly, cell death was not observed before the transition of α-synuclein aggregates to an LP-like form and was only modest afterward [55].

Lastly, it is important to mention that α-synuclein might be our best bet for the early detection of PD. Protein misfolding cyclic amplification, a technique to amplify the conformational state of α-synuclein from patient samples, has recently been utilized to detect α-synuclein aggregates in cerebrospinal fluid samples [56] with high sensitivity. Since misfolded α-synuclein has been reported in peripheral tissues [57], there is hope that it could serve as a marker for early detection of PD.

### How do dopamine neurons die?

The current understanding of molecular pathways leading to dopamine neuron degeneration reveals a complex, often reciprocal interaction between specific physiology of dopamine neurons, oxidative stress and mitochondrial dysfunction, proteostatic stress, disruption of autophagy–lysosomal pathway and accumulation of misfolded α-synuclein. Strong evidence links all these processes with dopamine neuron degeneration in PD [47]. However, we are missing the vital piece of the puzzle, which is understanding how dopamine neurons die in PD. Major cell death types include apoptosis, necrosis and autophagic cell death [58]. Apoptosis and autophagic cell death are classically programmed events, however in many cases it is now clear that necrosis can also be regulated to some extent by cells. Therefore, cell death is usually not just a catastrophic breakdown but orchestrated sequence of events that serves the survival of the whole organism [58, 59]. This gives hope that neuron demise can be delayed or prevented by blocking specific cell death pathways, at least before the cell reaches a point of no return such as mitochondrial membrane permeabilization [58].

Because of the dynamic nature of the cell death process and only transient activation of involved pathways [59], distinguishing different cell death types is difficult in post-mortem samples. Nonetheless, many, but not all, studies on post-mortem samples revealed signs of apoptosis such as DNA damage and elevated activity of caspases, which are cysteine-containing proteases that cleave substrates after aspartic acid [60]. However, most of the data supporting the involvement of apoptosis in PD comes from preclinical cellular and animal models [60]. Findings from neurotoxin-based models revealed a critical role of intrinsic mitochondrial apoptosis pathways in dopamine neuron death. The relevance of this pathway for humans is supported indirectly by the fact that genes linked with familial PD, namely DJ-1, PINK1, and Parkin, were shown to regulate the mitochondrial apoptosis pathway in neurotoxin models [60]. Apart from toxins damaging mitochondria, it was also proposed that apoptosis in dopamine neurons can be initiated by prolonged activation of the UPR due to the accumulation of misfolded proteins and DNA damage through the p53-dependent pathway [60].

The involvement of autophagic cell death and necrosis in dopamine neuron death in PD is less clear than the involvement of apoptosis. There are reports on autophagy in dying neurons in PD patients' brains and neurotoxic models [60], however it is unclear if they account for autophagic cell death or might actually be the last attempt of a cell to prevent its demise [59]. Evidence for the involvement of necrosis comes from preclinical studies, where the same neurotoxins that induce apoptosis at low and moderate doses cause necrosis when administered at high doses [60].

Ultimately, it is important to remember that the majority of specific data on the death pathway of dopamine neurons comes from cellular and animal models under the assumption that they resemble cell death pathways of human PD patients.

### Can a current understanding of molecular pathology in PD guide us toward new therapies?

There is strong evidence for the involvement of all mentioned processes in the death of dopamine neurons in PD. Multiple trials targeting specific mechanisms are underway (Table 1), so far however, such treatments have failed. The current state of knowledge does not allow us to find reasons for this failure unequivocally, but several options could be considered. Multiple pieces of evidence support the involvement of oxidative stress and mitochondrial failure, autophagy-lysosomal pathway disruption, proteostasis disruption, ER stress, and α-synuclein misfolding and aggregation in PD pathology. Moreover, data shows that these cellular pathologies are interconnected, capable of inducing or aggravating each other, possibly causing a cascade of failures, as has also been proposed by other authors [12, 61–63]. However, current data do not clearly point to any specific pathological process as the main trigger of pathology nor as the main cause of dopamine neuron death. In fact, it is possible that no single mechanism can be pinpointed because no single mechanism for all patients [21], nor even maybe for all neurons in a single patient, exists. Because what we are observing are relatively downstream phenotypes, multiple pathways could exist to reach dopamine neuron death. From a therapeutic perspective, it means that drugs targeting pathological triggering mechanisms would have to be personalized for a given patient, basing treatment on yet unknown markers. However, current and past clinical trials for PD have been mostly targeting general patient populations, and this has been proposed as a reason for failure [21]. Moreover, even if a single mechanism is triggering pathology for most patients, we would need to target it before it causes a cascading failure of other cellular systems. In contrast to mechanism-specific treatments, general anti-apoptotic (assuming apoptosis as major cell death mechanism in PD) or survival-promoting treatments like NTFs, should be effective regardless of initial trigger and subsequent path of pathology progression (Fig. 1), as discussed below.

**Table 1:** List of clinical trials targeting described pathological processes linked with dopamine neuron degeneration

Putative mechanism	Treatment	Description	Trial identifier or reference	Trial phase	Trial status
Preventing α-synuclein accumulation	ABBV-0805	Antibody against α-synuclein	NCT04127695	1	Pre-recruitment
	AFFITOPE PD01/PD03	Vaccine against α-synuclein	NCT02618941, NCT02267434	1	Completed
	MEDI1341	Antibody against α-synuclein	NCT03272165	1	Recruiting
	BIIB054	Antibody against α-synuclein	NCT02459886	1	Completed
	Prasinezumab	Antibody against α-synuclein	NCT03100149	2	Active
	Lu AF82422	Antibody against α-synuclein	NCT03611569	1	Recruiting
	UB-312	Vaccine against α-synuclein	NCT04075318	1	Recruiting
	Phenylbutyrate	Small molecule increasing removal of α-synuclein from brain	NCT02046434	1	Active
	Mannitol	Small molecule, disrupts BBB and increase removal of α-synuclein from brain. Iinhibit α-synuclein aggregation	NCT03823638	2	Recruiting
	NPT200-11	Small molecule inhibitor of α-synuclein misfolding	NCT02606682	1	Completed
Reducing mitochondrial dysfunctions	Deferiprone	Small molecule iron chelator	NCT02655315	2	Recruiting
	CNM-Au8	Gold nanoparticles improve mitochondrial function, antioxidant	NCT03815916	1	Recruiting
	CU(II)ATSM	Small molecule improve mitochondrial function, antioxidant	NCT03204929	1	Active
	UDCA	Small molecule, ursodeoxycholic acid, improve mitochondrial function	NCT03840005	2	Recruiting
Targeting PD linked genes	BIIB094	Antisense oligonucleotide LRRK2	NCT03976349	1	Recruiting
	DNL-151	Small molecule inhibitor of LRRK2	NCT04056689	1	Recruiting
	DNL-201	Small molecule inhibitor of LRRK2	NCT03710707	1	Active
	Ambroxol	Small molecule enhancer of GBA activity	NCT02941822	2	Active
	PR001A	Gene threrapy, expression of functional GBA	NCT04127578	1/2	Recruiting
	GZ/SAR402671	Small molecule enhancer of GBA activity	NCT02906020	2	Recruiting
Neuroprotection and reduced inflammation	Exenatide	Peptide agonist of GLP-1	NCT03456687	3	Recruiting
	Semaglutide	Peptide agonist of GLP-1	NCT03659682	2	Pre-recruitment
	Liraglutide	Peptide agonist of GLP-1	NCT02953665	2	Recruiting
	Lixisenatide	Peptide agonist of GLP-1	NCT03439943	2	Recruiting
	NLY01	Peptide agonist of GLP-1	NCT04154072	2	Pre-recruitment
	NPT520-3	Small molecule SLC22A8 inhibitor, reduce nueroinflammation	NCT03954600	1	Recruiting
Neurotrophic action	AAV2-GDNF	Gene therapy, GDNF expression. Neurotrophic effects	NCT04167540	1	Recruiting
	AAV2-GDNF	Gene therapy, GDNF expression. Neurotrophic effects	NCT01621581	1	Active
	NTCELL	Encapsulated choroid plexus cells, produce neurotrophic factors	NCT01734733	1/2	Active
	ITI-214	Small molecule PDE1 inhibitor, increase cAMP levels, might increase NTFs production and function	NCT03257046	1/2	Completed
	PDGF-BB	Direct infusion of growth factor PDFG-BB,	NCT02236793	3	Ineffective
	GDNF	GDNF protein, monthly boluses to ventricle	Nutt et al. [128]	1/2	Completed, see text
	GDNF	GDNF protein, continuous infusion to putamen	Gill et al. [129]	1	Completed, see text
	GDNF	GDNF protein, continuous infusion to putamen	Slevin et al. [130]	1	Completed, see text
	GDNF	GDNF protein, continuous infusion to putamen	Lang et al. [133]	2	Completed, see text
	GDNF	GDNF protein, boluses to putamen	NCT03652363	2	Completed, see text
	AAV2-NRTN	Gene therapy, NRTN expression in putamen. Neurotrophic effects	NCT00252850	1	Completed, see text
	AAV2-NRTN	Gene therapy, NRTN expression in putamen. Neurotrophic effects	NCT00400634	2	Completed, see text
	AAV2-NRTN	Gene therapy, NRTN expression in putamen and SN. Neurotrophic effects	NCT00985517	1	Completed, see text
Neurotrophic and other actions	CDNF	Protein infusion, general neuroprotective effects, axon regrowth, interferes with α-synuclein oligomerization, reduces ER stress	NCT03295786	1/2	Active
Stimulate autophagy-lyzosomal pathway	Nilotinib	Small molecule c-Abl inhibitor, enhances autophagy	NCT03205488	2	Active, ineffective
	K0706	Small molecule c-Abl inhibitor, enhances autophagy	NCT03655236	2	Recruiting
Reduce Ca^2+^ fluctuations	Isradipine	Small molecule calcium channel blocker, reduces calcium fluctuations	NCT02168842	3	Active, ineffective
Blocks apoptosis	KM-819	Small molecule FAF1 inhibitor, inhibits apoptosis	NCT03022799	1	Completed

Special consideration should be given to the treatments targeting α-synuclein. We still do not know if α-synuclein is a causative factor in non-familial PD nor do we know mechanisms by which it contributes to cell death. Understanding the putative mechanism through which α-synuclein contributes to cell death is critical since it impacts which treatments might be effective, and which could even worsen disease progression. For example, we do not know if α-synuclein early oligomeric forms or rather Lewy bodies should be targeted. If Lewy bodies are a sink for toxic oligomeric forms, their disruption could seal the fate of neurons. Growth of α-synuclein fibrils is also not sufficiently understood. If secondary nucleation is important in this process, treatments, such as antibodies capping α-synuclein, might actually promote the formation of smaller and presumably more toxic species through secondary nucleation. Nonetheless, several anti-α-synuclein antibodies and small molecule inhibitors of α-synuclein aggregation are currently tested in the clinic (Table 1). Drawing parallels to another neurodegenerative disorder—Alzheimer's disease—where targeting pathological protein aggregates have consistently failed in the clinic [64], targeting LP for the treatment of PD should be preceded by more systematic basic studies on the pathogenic mechanisms, because clinical trials planned on the basis of insufficient information may easily fail and discourage from pursuing this otherwise promising approach. Indeed, a recent press release about a clinical trial of Prasinezumab (NCT03100149), a humanized monoclonal antibody against α-synuclein, reported that the study did not meet a primary objective (https://ir.prothena.com/news-releases/news-release-details/update-phase-2-pasadena-study-prasinezumab-prx002rg7935).

## Neurotrophic factors in preclinical studies

**Fig. 3:**
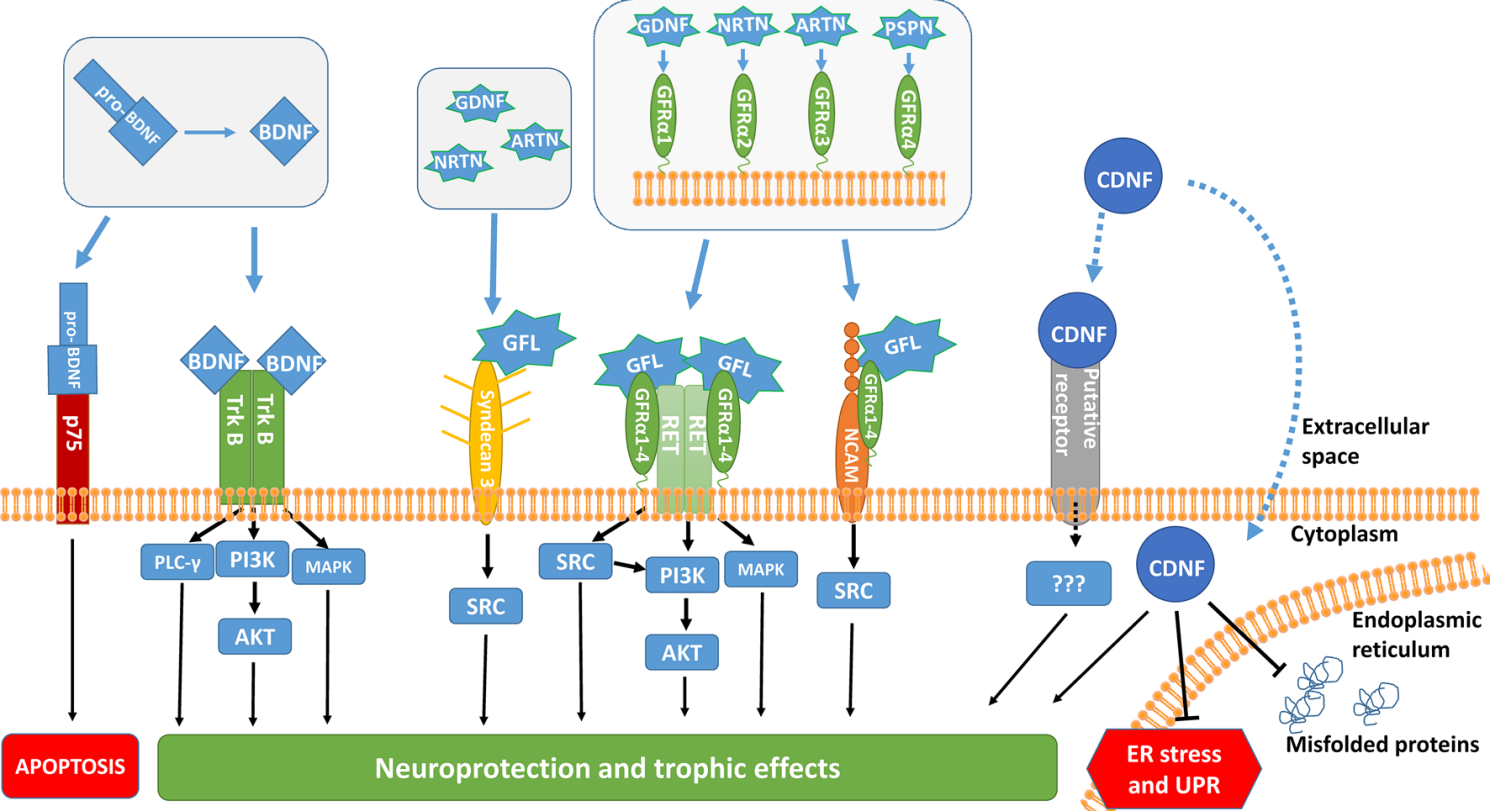
Receptors and signaling modes of brain-derived neurotrophic factor (BDNF), glial cell line-derived neurotrophic factor (GDNF)-family ligands (GFLs) and cerebral dopamine neurotrophic factor (CDNF). The mature form of BDNF exerts its neuroprotective effects through tyrosine kinase receptor B (TrkB), activating mitogen-activated protein kinase (MAPK), phosphatidylinositol-4,5-bisphosphate 3-kinase (PI3K)/Akt and phospholipase C-γ pathways (PLCγ) pathways. Alternatively, pro-BDNF can exert pro-apoptotic effects through p75 receptor. GFLs: GDNF, NRTN, ARTN and PSPN, act mainly through receptor tyrosine kinase RET together, requiring additional coreceptors GDNF Family Receptor alpha 1 to 4 (GFRα1-4). GDNF, NRTN, ARTN and PSPN bind to GFRα1 to 4, respectively. Binding of GFL-GFRα complex to RET activates MAPK, Src and PI3K/Akt signaling pathways. Additionally, GFLs can also signal through GFRα-NCAM and Syndecan 3 (with the exception of PSPN in the latter case). CDNF is an unconventional neurotrophic factor without a known membrane receptor. Putatively, CDNF can act both on the plasma membrane and intracellularly on the ER membrane. It exerts prosurvival effects attenuating ER stress and interacting with misfolded proteins

The rationale for NTF-based treatments of PD is their ability to both support the survival, regenerate axons, and increase neuronal function and connectivity. Therefore, they could not only protect remaining dopamine neurons but also stimulate their regeneration and capacity to make up for already lost cells. NTFs are secretory molecules important in neuronal development, maintenance, and synaptic plasticity. Therefore, NTFs with the ability to promote the survival of dopamine neurons have been tested extensively in preclinical models of PD. We have recently summarized the strengths, limitations, and future perspectives of PD models in a different review [65]. Most promising NTFs have been tested or are being tested in clinical trials. Probably the best-studied NTFs in the context of PD are glial cell line-derived neurotrophic factor (GDNF) and another GDNF-family ligand neurturin (NRTN), brain-derived neurotrophic factor (BDNF), CDNF and mesencephalic astrocyte-derived neurotrophic factor (MANF) (Fig. 3).

### GDNF and GDNF-family ligands

GDNF is an eponymous member of the GDNF family ligands (GFLs), which also consists of NRTN, artemin (ARTN) and persephin (PSPN) [8]. GDNF was first described in the early nineties as a factor promoting survival of cultured midbrain dopamine neurons [66], thus not surprisingly, it was quickly tested as a potential therapeutic molecule for PD. GFLs are distant members of the transforming growth factor β superfamily [8], and differently from other members of this family, GFLs signal through receptor tyrosine kinase RET [67]. However, this signaling requires coreceptors: GDNF Family Receptor alpha 1 to 4 (GFRα1-4). GFRα1-4 are usually glycosyl phosphatidylinositol (GPI) anchored at the plasma membrane or after cleavage of the GPI anchor are also present in a soluble form. GFLs do not bind directly to RET, but first to GFRα coreceptor and then the GFL-GFRα complex binds to and activates RET [67, 68]. GFRα1-4 determines the ligand selectivity of GDNF, NRTN, ARTN, and PSPN [8, 67, 68]. Binding of GDNF to GFRα1 and then to RET leads to RET dimerization, tyrosine autophosphorylation and activation of phosphatidylinositol-4,5-bisphosphate 3-kinase (PI3K)/Akt, mitogen-activated protein kinase (MAPK) and Src pathways [8] (Fig. 3, middle). Additionally, GDNF and other GFLs can also bind to and signal through Neural Cell Adhesion Molecule (NCAM) activating Src pathway [69] (Fig. 3, middle). GDNF, NRTN, and ARTN can also bind and signal through heparan sulphate proteoglycan Syndecan-3 present at the cell surface [70] (Fig. 3, middle). RET and GFRα1 are expressed on SNpc dopamine neurons, however the main physiological site of GDNF action is at their projections in the striatum [67]. GDNF infusions have been consistently demonstrated to be effective in animal neurotoxin models of PD [71]. It rescued 6-hydroxydopamine (6-OHDA)-induced lesions in rodents [72] and in 1-methyl-4-phenyl-1,2,3,6-tetrahydropyridine (MPTP) both in rodents [73] and non-human primates [74]. Importantly, GDNF was effective even when administered several weeks after neurotoxin treatment, demonstrating at least some potential at restoring already damaged dopamine neurons and their function [75]. NRTN, another GFL, has been shown to be similarly protective as GDNF in animal models of PD [76]. Both GDNF and NRTN were protective when administered as either a protein or expressed from viral vectors [77, 78]. Its effectiveness in animal models led to a quick move of GDNF to clinical trials, findings of which will be summarized in the following section. It should be noted, however, that GDNF and NRTN were effective in mild and moderate neurotoxin models, whereas they showed weak or no neuroprotection when the nigrostriatal pathway lesion was severe. Furthermore, it was shown that GDNF failed to protect dopamine neurons from cell death induced by α-synuclein overexpression [79, 80]. This lack of effectiveness was presumably caused by the downregulation of RET and transcription factor Nurr1, both important for GDNF’s protective effects [81]. However, it remains disputed if findings from this animal model, where α-synuclein was overexpressed at high levels, are relevant for human patients [82].

### BDNF

BDNF is a member of the neurotrophin family, originally described in the 1980s [83]. BDNF acts on cells by binding to either tyrosine kinase receptor (Trk) B or to low-affinity p75 neurotrophin receptor (p75), a member of tumor necrosis factor receptor superfamily [84]. BDNF can also bind to the TrkB-p75 complex. Survival promoting effects of BDNF are mediated by the TrkB receptor through activation of MAPK, Akt, and phospholipase C-γ pathways [84], while p75 receptors are mainly activated by BDNF precursor molecule, proBDNF triggering pro-apoptotic activity [84] (Fig. 3, left). Interestingly, p75 seems to be upregulated in neurodegenerative disorders and aging [85]. In human PD patients, BDNF levels were found to be decreased in the SNpc [86] and in the serum [87]. However, a quite surprising increase in the BDNF levels in later stages of the disease was also reported [88]. BDNF promotes the survival of dopamine neurons in vitro and in animal models of PD [89–91]. However, in contrast to GDNF and CDNF, BDNF was effective only when administered prior to neurotoxin, what suggest it would have to be administered at very early stages of PD. Altogether, it’s reasonable to assume that BDNF, while promising, will require the development of non-invasive administration strategies to very early stage patients as administering it to later-stage patients is currently not supported by preclinical evidence. Conversely, despite its links to PD and encouraging preclinical data supporting BDNF has not been tested in the clinic for the treatment of PD.

### CDNF and MANF

CDNF and MANF form a novel, evolutionary conserved family of unconventional NTFs [92, 93]. Both proteins are paralogs with similar structures, different in amino acid sequence and in three-dimensional structure from all other NTFs [94]. They reside in the ER, where they play a role in maintaining protein homeostasis, and can be secreted in a calcium-dependent manner [95], but are also secreted in stress or cell injury. However, when expressed in cells or applied externally to stressed cells, they exert survival-promoting effects on neurons and attenuate ER stress and the UPR [94] (Fig. 3, right). The mechanism of their action is still unknown, as are their putative receptor(s). Both MANF and CDNF were shown to be protective in vivo in 6-OHDA and MPTP rodent models of PD, both when administered as protein and when overexpressed from viral vectors [92, 96–99]. Moreover, CDNF was shown to be effective in the non-human primate 6-OHDA model of PD [100]. The neuroprotective potential of CDNF and MANF has been summarized in recent reviews [101, 102]. Overall, CDNF is at least as effective as GDNF [103] with the important advantage of having better diffusion through the brain [96], and in addition to inhibiting neuronal apoptosis, also regulating ER stress and reducing the levels of pro-inflammatory cytokines. Better bioavailability would be important when infused into the human brain, much larger than that of rodents. Efficacy, desirable properties (diffusion) and a novel mechanism of action combining survival-promoting action with positive effects on protein homeostasis made CDNF an exciting new candidate for PD treatment. Indeed phase I-II clinical trial of CDNF in PD patients was recently initiated in three medical centers [102] (NCT03295786).

### Importance of NTFs for dopamine neurons

While NTFs show protective effects on dopamine neurons, and may be required for their differentiation, it is not entirely clear whether they are necessary for dopamine neuron survival in normal physiological conditions. For example, BDNF and its receptor TrkB are not required for the survival of dopamine neurons, but BDNF plays a role in their maturation [104]**.** As for GDNF, probably the most potent and most studied survival promoting NTFs for dopamine neurons, there have been some conflicting results about its necessity for adult dopamine neuron survival [105–107], whereas GDNF receptor RET is widely accepted to be necessary for their adult maintenance [108]. Interestingly, MANF knockout mice show a normal number of nigrostriatal dopamine neurons and their fibers [109] but in worms MANF seems to be required for long term maintenance of dopamine neurons [110]. Also, the MANF-deficient fly [111] and zebrafish [112] show defects in the dopamine neuron system. Analysis of the CDNF-deficient mice revealed that the number of dopamine neurons in SN and dopamine and its metabolite concentrations in the striatum are unaltered. However, an age-dependent functional deficit in the dopamine system function was found in Cdnf^−/−^ mice [7]. These mice develop an age-dependent loss of enteric neurons occurring selectively in the submucosal but not in the myenteric plexus resulting in delayed gastric emptying, slowed colonic motility, and prolonged total gastrointestinal transit. The deficiencies of Cdnf^−/−^ mice, therefore, are similar to those seen in early stages of PD.

### How do NTFs compare to other treatment strategies?

NTFs exert survival-promoting effects on dopamine neurons in diverse models, with at least some capability of promoting regeneration of axons and restoring dopaminergic phenotype. They seem to act through activating major pro-survival and anti-apoptotic pathways like MAPK, Src and AKT pathways. Consequently, NTFs have the greatest chance to work regardless of which pathological process is the major driver in a given patient, and should not require patient stratification. Moreover, as they can exert anti-apoptotic action relatively downstream of pathology, they could also work at stages where blocking a single pathological process (i.e. oxidative stress) is not sufficient (Fig. 1). The anti-apoptotic action of NTFs might not be enough to save dopamine neurons in PD since it is not unequivocal if apoptosis is the main cell death mode in PD. It is plausible that if NTFs block apoptosis, it will prolong the survival of neurons, but the damage would accumulate, and at some point, the neurons will die by non-apoptotic death. Nonetheless, prolonging survival of remaining neurons would still be of great benefit. If administered early after diagnosis, NTFs could prolong survival of the remaining 40–60% of dopamine cells which are still present at the onset of motor symptoms. Additionally, NTFs promote regeneration and function of dopamine cells. This could restore function in some neurons which presumably lost their dopamine phenotype but are still alive. Moreover, increasing the function of remaining neurons would compensate for lost ones. The beneficial effects would probably be even more pronounced if applied during the pre-symptomatic stage. Conversely, it is doubtful that NTFs will exert significant clinical benefit when administered to late-stage patients with only a small fraction of dopamine neurons left. Additionally, there are some reports that NTFs could have a dual action—both survival-promoting and affecting a specific pathological mechanism. Namely, CDNF can attenuate ER stress and regulate protein homeostasis. The effects of NTFs on specific pathologies would probably be most beneficial if applied very early in disease progression.

On the other hand, the effect of NTFs on non-motor symptoms might be limited. Effects of NTFs on the degeneration of non-dopaminergic neurons in PD will depend on both the presence of specific receptors and delivery mode. The most important GDNF receptor RET has limited expression in the brain, however, it is present on noradrenergic neurons, which are also profoundly affected in PD. BDNF and CDNF, in contrast, can affect more diverse neuronal populations. However, at present, delivery strategies aim at targeting dopamine neurons. In the future, blood–brain barrier permeable variants of NTFs or NTFs mimicking compounds might allow a peripheral delivery method. This would have both advantages of being acceptable to early-stage patients or pre-symptomatic patients as well as affecting multiple cellular populations.

### Different approaches to stimulate NTF-linked pathways

NTFs have mostly been applied either as a direct protein infusion or expressed from viral vectors. However, both of these approaches have significant drawbacks. Described NTFs do not pass through the blood–brain barrier, therefore requiring complicated stereotactic brain surgery to administer. At the same time, their diffusion in the brain is limited, and the production of NTF-based drugs can be costly [77]. Viral overexpression of NTFs allows continuous treatment without a need for repetitive injections. However, NTF gene therapy also requires a complicated intracranial administration. Apart from complicating clinical trials, the risk involved in surgical administration has played a role in limiting NTF clinical trials to relatively late-stage patients due to ethical concerns.

Several alternative approaches to protein or viral vector administration have been pursued to realize the therapeutic potential of NTFs. Continuous delivery of NTFs into the brain could be achieved with encapsulated cell (EC) technology [113]. EC is based on the implantation of cells enclosed in semi-permeable membranes, which protect them from the host immune system while allowing them to release NTFs into the surrounding tissue and absorb nutrients. Such cells can be genetically engineered to secrete high levels of desired protein with the additional possibility of adding regulatory mechanisms, which would be difficult to fit into viral vectors. Moreover, EC devices can be designed so that it is possible to remove them from the brain in case of adverse reactions. The use of EC implants also avoids problems with the transduction efficiency of viral vectors in the aged brain, which was recently implied for AAV2 vectors [114]. Nonetheless, while EC implants still have to be delivered through a surgical procedure, and unless NTFs with good diffusion properties are utilized, they might require several deposition sites for NTFs to reach the entire putamen. Preclinical studies already demonstrated highly efficient delivery of GDNF by genetically engineered human ARPE-19 cells in rat striatum [115]. Also, ARPE-19 cells expressing CDNF, which have the advantage of better diffusion in the brain, were recently characterized by us [116]. Importantly, ARPE-19-based EC implants have already demonstrated robust survival when implanted into Alzheimer patients’ parenchyma, demonstrating clinical maturity of the EC technology [113]. Another obvious (but not necessarily trivial) approach is to use small molecule agonists of NTF receptors (NTF mimetics) [117, 118]. A blood–brain barrier permeable GDNF mimetic demonstrated protective effects on dopamine neurons both in vitro and in vivo [118]. It is also possible to induce endogenous production of NTFs either through small molecules or interventions such as physical exercise [119]. Indeed, stimulation of BDNF production could explain some positive effects of physical exercise in PD [120]. Lastly, activating transcription factors downstream of NTFs, and putatively mediating their survival-promoting effects, could have therapeutic potential. It was shown that overexpression of Nurr1, which induces RET expression and is activated by GDNF, is protective for dopamine neurons [121]. Interestingly, several reported small molecule activators target Nurr1, and could potentially be used in the clinic [122]. Nonetheless, while promising, these approaches have not yet been extensively validated, and (apart from intervention exercise), tested in the clinic for PD treatment. Approaches for the administration of NTFs or activation of NTF-linked pathways have been summarized in Table 2.

**Table 2:** Overview of described methods to engage NTF protective pathways in PD

Method	Advancement stage	Delivery difficulty	Action time	Other advantages/disadvantages	References
Direct NTF infusion	Tested in clinic	Complicated (initial implantation)	Transient (monthly injections)	Best proven in preclinical studies, difficult production	[71–76, 92, 96, 99, 100, 123–132]
Viral NTF delivery	Tested in clinic	Complicated	Lasting	Widely tested in preclinical studies risk with viral transduction, difficult production limited ability to cease treatment limited transduction efficiency in aged brain	[77, 78, 97, 98, 133, 134]
Encapsulated cells releasing NTFs	High	Complicated	Lasting	Constant delivery, good coverage, potential to engineer regulatory mechanisms, complicated production	[113, 115, 116]
Small molecule NTFs mimetics	High	Easy	Transient (daily or weekly?)	Suitable for early stage patients, excellent coverage, possible side effects due to action outside brain	[117, 118]
Induction of endogenous NTFs	Medium/in clinic (physical exercise)	Depends on method (easy for pharmacological or lifestyle interventions)	Depends	Possibly less side effects (physiological site of action), might depend on disease stage (presence of cells able to produce NTFs)	[119, 120]
Transcription factor therapy	Early	Easy (small molecules), complicated (gene therapy)	Transient (small molecules), lasting (gene therapy)	Independent from NTFs receptors, advantages of small molecule mimetics, might require gene therapy	[121, 122]

## Neurotrophic factors in clinic

Due to promising preclinical evidence, several NTFs entered clinical trials for PD (Table 2), despite complications linked with their application in humans and uncertain mechanisms of action on brain dopamine neurons. However, complications linked with the delivery and insufficient understanding of disease etiology might have led to sub-optimal trial design and not meeting clinical endpoints.

### GDNF

The first clinical trials for GDNF in PD started almost two decades ago. In these trials, GDNF was given monthly for over 8 months to 50 patients in a double-blind controlled trial design [123]. To simplify the delivery procedure, the drug was administered into the lateral ventricle, retrospectively leading to concerns if GDNF was able to reach the site of action, considering its poor diffusion in the brain. No clinical improvement was observed, and even worse, at very high GDNF doses, side effects like nausea and weight loss were observed in some patients. Side effects were also attributed to a non-physiological site of delivery. In the subsequent trials, GDNF was delivered into the brain parenchyma, specifically into caudate putamen, correspondingly to preclinical data. Two small open-label studies were made on 5 PD patients [124] and 10 PD patients, respectively [125, 126]. In these trials, GDNF demonstrated 30–60% improvement in scores for motor activity and daily living. Improvements were accompanied by increased markers of dopamine neuron function in the putamen, as assessed by PET [124]. Also, an increase in dopaminergic innervation was found in one post-mortem patient autopsy [127]. Following positive results in these two open-label trials, new placebo-controlled clinical trials were started [128]. GDNF in this new trial was administered continuously into the caudate putamen via the infusion pump. However, after 6 months of treatment, no difference from the placebo group was observed. Moreover, very surprisingly, some patients developed neutralizing antibodies for GDNF, which led to study withdrawal from safety concerns. Interestingly, one of the patients showed persistent benefits 3 years after treatment cessation, suggesting effectiveness, at least in a subpopulation of patients [129]. It remains currently unclear how GDNF neutralizing antibodies could have arisen from intraparenchymal infusions of the protein. Results from another placebo-controlled trial were reported recently [130, 131]. There, GDNF was administrated intraputamentally with a convection-enhanced delivery system once in a month for 6 months. While no significant improvement in UPDRS scores was observed, the study demonstrated the safety of GDNF infusion and an increase in dopamine neuron function in all GDNF treated patients, assessed through PET imaging. In line with the GDNF-induced increase in dopamine neuron activity, a post-hoc analysis found nine (43%) patients receiving GDNF, but not placebo patients, demonstrated large motor improvements in the OFF state. Lastly, an open-label gene therapy study with 25 patients receiving AAV2-GDNF vector into the putamen is currently ongoing (NCT01621581).

### NRTN

NRTN was tested as a gene therapy in three clinical trials for PD. Initial open-label trials with intraputamental infusion showed some improvements in UPDRS, but not in PET markers [132]. However, two subsequent double-blinded studies, one with putamental delivery [133] and the other one where AAV2-NRTN was delivered simultaneously into both putamen and to SN [134] failed to meet clinical endpoints. Nonetheless, when patients were stratified by time from disease diagnosis, some benefits were observed in earlier stage PD patients [133, 135]. The long-term effects of AAV-NRTN injections were recently described in a post-mortem study of two patients 8 and 10 years after the virus injection [114], the former receiving injections to both putamen and SN and the latter to putamen only. The observed NRTN expression was limited to 3–12% coverage in the putamen, and 9–40% in SN, which probably contributed to the lack of clinical benefit. Significantly, in the areas where NRTN was expressed, it strongly increased dopaminergic innervation and dopamine cell markers, in putamen and SN, respectively, demonstrating long term benefits of NRTN. This indicates the capability of NRTN to protect and restore the function of dopamine neurons over a span of almost a decade. In the SN many protected neurons were positive for α-synuclein aggregates, suggesting that NRTN can protect neurons affected by pathological processes in PD. However, saving dopamine neurons in the SN have not improved innervation of the putamen, and conversely, administration of NRTN in putamen had only limited effects on the survival of dopamine neurons in the SN. This might be because at the time of administrations which was > 5 years after PD diagnosis, surviving dopamine cells in the SN might have already lost connection to the putamen, either due to degeneration of axons or disturbed axonal trafficking.

### CDNF

The double-blind clinical phase I/II trials for CDNF therapy for PD was started in autumn 2017 [102]. Importantly, CDNF therapy has the potential to have better coverage thanks to methodological improvements based on previous NTF studies and better brain diffusion of the protein. CDNF is delivered by a convection-enhanced delivery system once a month for 6 months, which will be followed by an open-label extension period where all patients will be given CDNF. The first results of the 6 months study were announced in early 2020. Initial data suggest that CDNF is safe and shows promising signals of biological activity e.g. in dopamine transporter PET imaging in some patients, supporting the hypothesis of CDNF’s disease-modifying potential. The treatments continue and further results are expected in autumn 2020 (https://herantis.com/press_releases/herantis-pharma-plc-announces-topline-results-of-phase-1-2-cdnf-trial/).

### Difficulties, limitations and possible solutions

None of the completed double-blind clinical trials for NTF administrations met predefined endpoints for clinical efficacy. The design of clinical trials for neuroprotection in PD remains challenging, conversely inadequate design might have contributed to the failure to demonstrate neuroprotection, as was discussed extensively by other authors [23, 136]. Furthermore, post-mortem data strongly suggest that technical difficulties in delivering NTFs either as protein or viral vector and poor diffusion of GDNF and NRTN in the brain resulted in limited coverage of the targeted area [114]. Moreover, failed double-blind trials were conducted in late state patients. According to our current knowledge of PD progression, this very significantly limited chances of the success of these trials. Conversely, post-hoc analyses showing more benefits in patients with less advanced PD [133] corroborate the notion that disease stage is a crucial factor contributing to chances of success.

Yet, despite failure to meet clinical endpoints, brain imaging and post-mortem data demonstrate the capability of NTFs to restore the function of the dopamine system and to protect dopamine neurons over the span of almost a decade in humans, despite clear signs of pathological processes inside protected cells [114]. This strongly supports the notion that NTFs, thanks to general survival- and function-promoting effects, can effectively rescue dopamine neurons even with advanced stages of pathology. However, dopamine neurons must still be present in sufficient numbers, most likely with intact or at least functional axonal projections to the putamen to achieve clinical benefit.

## Conclusions

Preclinical data strongly suggest complicated and possibly self-aggravating pathology in multiple intracellular processes [12, 61–63]. Moreover, it seems plausible that the course of the disease differs between patients and might require drastically different treatments for different patient populations, targeting a patient-specific pathological pathway [21]. Therefore, slowing PD progression with drugs targeting specific mechanisms might be possible only with the personalized approach at the very early, presymptomatic stages of PD. So far none such single mechanism targeting drugs has demonstrated protective effects. In contrast, MAO inhibitors which have multiple mechanisms of action, including general survival-promoting effects, possibly through NTF-linked pathways, demonstrated hints of disease-modifying effects in double-blind clinical trials [22]. Conversely, NTFs with their general survival- and function-promoting effects on dopamine neurons could be effective, despite our lack of full understanding of disease etiology and without the need for patient-specific treatments. As discussed in this article, NTFs have effectively protected dopamine neurons in multiple preclinical PD models and showed some potential to restore the function of damaged dopamine neurons and their axons. Moreover, despite failing to meet predesigned endpoints for clinical improvement, NTFs were able to enhance some aspects of dopamine system function and were able to improve innervation and increase survival of dopamine cells for almost a decade in PD patients [114, 135]. Taking it all into account, failing to meet clinical endpoints does not invalidate NTFs as a treatment for PD but rather points toward technical challenges which limited the clinical benefit of treatment. These technical obstacles have already been discussed by other authors [4, 102], and include difficulties with the administration of proper dose, covering a large brain area, and recruitment of early-stage patients—for which not only a safe but also minimally invasive administration method would be optimal. Additionally, conceptual redesign of clinical trials might help to detect even modest sings of NTFs’ effectiveness in late-stage patients, which in turn would help to convince regulatory bodies for trials in early-stage patients [23]. Importantly, solving these difficulties seem to lie within our current capabilities rather than requiring scientific breakthrough. Altogether, despite setbacks and remaining technical difficulties, NTFs remain front-runner candidates for disease-modifying therapies of PD.

## Abbreviations

6-OHDA: 6-Hydoxydopamine
ARTN: Artemin
ASK1: Apoptosis signal-regulating kinase 1
ATF4: Activating transcription factor 4
ATF6: Activating transcription factor 6
BCL-2: B-cell lymphoma 2 anti-apoptotic protein
BDNF: Brain-derived neurotrophic factor
BiP: Binding immunoglobulin protein
Caspases: Cysteine-containing proteases that cleave substrates after aspartic acid
CDNF: Cerebral dopamine neurotrophic factor
CHOP: C/EBP homologous protein
eIF2A: Eukaryotic translation initiation factor 2A
EC: Encapsulated cell
ER: Endoplasmic reticulum
GBA: β-Glucocerebrosidase
GDNF: Glial cell line-derived neurotrophic factor
GFRα (1–4): GDNF Family Receptor alpha (1–4)
GFLs: GDNF family ligands
GPI: Glycosylphosphatidylinositol
IRE1α: Inositol-requiring protein 1
LP: Lewy pathology
MANF: Mesencephalic astrocyte-derived neurotrophic factor
MAPK: Mitogen-activated protein kinase
MPTP: 1-Methyl-4-phenyl-1,2,3,6-tetrahydropyridine
NCAM: Neural Cell Adhesion Molecule
NF-κB: Nuclear Factor Kappa-light-chain-enhancer of activated B cells
Nrf2: Erythroid 2-related factor 2
NRTN: Neurturin
NTFs: Neurotrophic factors
PD: Parkinson’s disease
PI3K: Phosphatidylinositol-4,5-bisphosphate 3-kinase
PERK: Protein kinase RNA-like ER kinase
PET: Positron emission tomography
PSPN: Persephin
PFFs: α-Synuclein preformed fibrils
SNpc: Substantia nigra pars compacta
SPECT: Single photon emission computed tomography
Trk: Tyrosine kinase receptor
UPR: Unfolded protein response
XBP1: X-box binding protein 1
